# A Novel Lactate Dehydrogenase Inhibitor, 1-(Phenylseleno)-4-(Trifluoromethyl) Benzene, Suppresses Tumor Growth through Apoptotic Cell Death

**DOI:** 10.1038/s41598-019-40617-3

**Published:** 2019-03-08

**Authors:** Eun-Yeong Kim, Tae-Wook Chung, Chang Woo Han, So Young Park, Kang Hyun Park, Se Bok Jang, Ki-Tae Ha

**Affiliations:** 1 Department of Korean Medical Science, School of Korean Medicine and Healthy Aging Korean Medical Research Center, Pusan National University, Yangsan Gyeongnam, 50612 Republic of Korea; 2 Department of Molecular Biology, College of Natural Science, Pusan National University, Geumjeong-gu Busan, 46241 Republic of Korea; 3 Department of Chemistry, College of Natural Science, Pusan National University, Geumjeong-gu Busan, 46241 Republic of Korea

## Abstract

The Warburg effect, wherein cancer cells prefer glycolysis rather than oxidative phosphorylation even under normoxic conditions, is a major characteristic of malignant tumors. Lactate dehydrogenase A (LDHA) is the main enzyme regulating the Warburg effect, and is thus, a major target for novel anti-cancer drug development. Through our ongoing screening of novel inhibitors, we found that several selenobenzene compounds have inhibitory effects on LDHA activity. Among them, 1-(phenylseleno)-4-(trifluoromethyl) benzene (PSTMB) had the most potent inhibitory effect on the enzymatic activity of LDHA. The results from biochemical assays and computational modeling showed that PSTMB inhibited LDHA activity. In addition, PSTMB inhibited the growth of several tumor cell lines, including NCI-H460, MCF-7, Hep3B, A375, HT29, and LLC. In HT29 human colon cancer cells, PSTMB dose-dependently inhibited the viability of the cells and activity of LDHA, without affecting the expression of LDHA. Under both normoxic and hypoxic conditions, PSTMB effectively reduced LDHA activity and lactate production. Furthermore, PSTMB induced mitochondria-mediated apoptosis of HT29 cells via production of reactive oxygen species. These results suggest that PSTMB may be a novel candidate for development of anti-cancer drugs by targeting cancer metabolism.

## Introduction

Most cancer cells show a unique metabolic preference for glycolysis rather than oxidative phosphorylation (OXPHOS), which is termed as the Warburg effect^1^. Although normal cells use glycolysis and lactic fermentation for ATP production only under low oxygen conditions, cancer cells employ these metabolic pathways even under high oxygen conditions^2^. This metabolic switch provides several advantages to cancer cells, i.e. fast ATP generation without reactive oxygen species (ROS) production, acidification of tumor microenvironment, and preservation of carbon building blocks for cell proliferation^1,3^. Thus, inhibition of this tumor-specific metabolism is a promising strategy for cancer treatment^4^. In most malignant cells, especially under hypoxic conditions, the expression of lactate dehydrogenase A (LDHA) is elevated via the hypoxia inducible factor 1 α (HIF-1α) and c-myc pathways^1,5,6^. In addition, LDHA directly converts pyruvate, a final product of glycolysis, to lactate^7^. For these reasons, among the several enzymes involved in glycolysis and lactic acid fermentation, LDHA is recognized as the key enzyme involved in the Warburg effect^8,9^.

Selenobenzene is a type of chalcogenide i.e. a chemical compound harboring at least one chalcogen anion and one more electropositive element^10^. The chalcogen elements, including oxygen, sulfur, and selenium, are constituents of the functional groups in biomolecules that are associated with redox chemistry^10,11^. Organic forms of selenium, such as diphenyl selenides and ebselen, exhibit antioxidant and cytoprotective effects by mimicking peroxidase activity^12,13^. Over the past decade, the construction of carbon-selenium bonds has remained an interesting topic for researchers, and there have been several publications reporting its therapeutic characteristics, such as their antimicrobial, antiviral, antioxidant, and antitumor properties^11^. Recently, we synthesized novel organochalcogenides by cross-coupling diphenyl diselenide and boronic acid through copper nanoparticle-catalyzed Se-Se bond activation^11^. Several previous reports demonstrated that diselenides show antitumor action through induction of apoptosis or inhibition of proliferation^14–16^. Thus, we hypothesized that these novel selenobenzenes may also have antitumor effects.

In this study, among various selenobenzenes that we tested, we found that 1-(phenylseleno)-4-(trifluoromethyl)benzene (PSTMB) has the most potent inhibitory effect on LDHA. The molecular mechanism underlying the LDHA inhibition and anti-tumor activity was investigated. From these results, we suggest that PSTMB can be a novel candidate for anti-tumor drug development by regulating cancer metabolism.

## Results

### *In Vitro* Evaluation of Inhibitory Action on LDHA Activity

**Figure 1:**
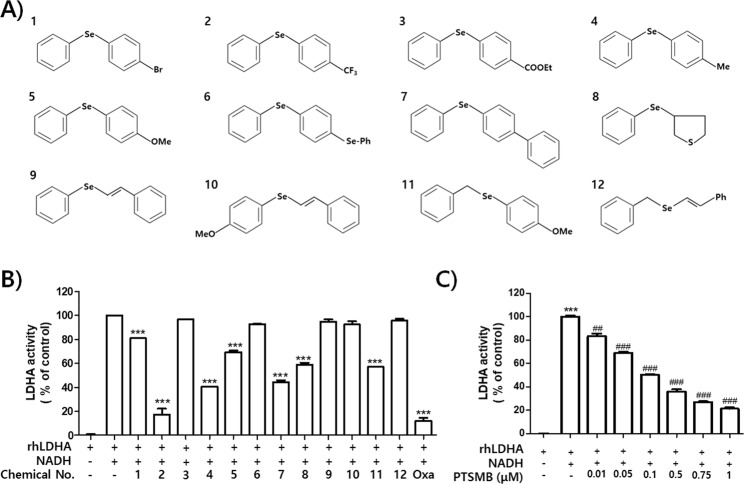
PSTMB has a potent inhibitory effect on *in vitro* LDHA activity. (**A**) Structures of the selenobenzene compounds analyzed in this study are shown. (**B**) The inhibitory activities of several selenobenzenes on LDHA activity were measured by *in vitro* LDHA assay using purified recombinant human LDHA. Oxamate (50 mM) was used as the positive control for LDHA inhibition. The results are presented as means ± SD. Data were statistically compared using the Student’s t-test. ***p < 0.001 compared to the positive control (2^nd^ column). (**C**) The dose-dependent inhibitory action of PSTMB on LDHA activity was examined using *in vitro* LDHA assay system. The results are presented as means ± SD. Data were statistically compared using one-way Analysis of Variance (ANOVA). ***p < 0.001 compared to the negative control (1^st^ column). ^###^*p* < 0.001 compared with the control (2^nd^ column).

Twelve selenobenzene compounds (Fig. 1A) were used in the *in vitro* LDHA activity assay. The result showed that PSTMB, 1-methyl-4-phenylselenobenzene, 1-methoxy-4-(phenylseleno)benzene, 4-(phenylseleno)-1,1′-biphenyl, tetrahydro-3-(phenylseleno) thiophene, and 1-methoxy-4-[(phenylmethyl)seleno]benzene had inhibitory effects on LDHA activity. These active compounds have not been reported as Pan Assay Interference Compounds (PAINS)^17^. Among these compounds, PSTMB showed the most potent inhibitory effect on LDHA activity (Fig. 1B). In addition, PSTMB showed dose-dependent inhibition of LDHA activity (Fig. 1C). The concentration at which PSTMB inhibits LDHA activity (IC_50_ = 145.2 nM) was much lower than that of oxamate (IC_50_ = 130.6 μM), a standard inhibitor of LDHA^18–20^.

**Figure 2:**
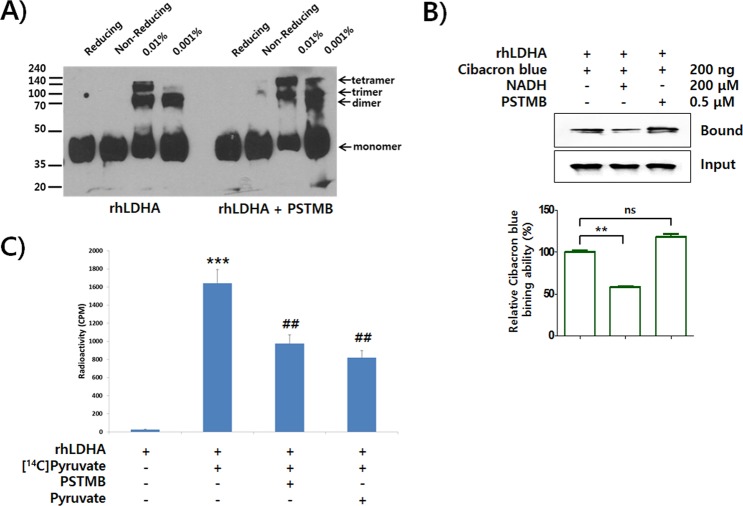
PSTMB inhibits pyruvate binding to LDHA. **(A)** The recombinant LDHA (rhLDHA) was incubated with or without PSTMB (0.5 μM). The samples were cross-linked (0.01% or 0.001% of final glutaraldehyde concentration) and fractionized by SDS-PAGE. The reducing and non-reducing samples are shown as a negative control. **(B)** The binding affinity of NADH toward LDHA was analyzed using Cibacron Blue as a mimicking probe of NADH. The rhLDHA was incubated with Cibacron Blue either in the absence or presence of PSTMB (0.5 μM). NADH was used as a competitor of Cibacron Blue binding. The LDHA bound to Cibacron Blue beads was size fractionated by SDS-PAGE, and evaluated by western blot analysis. The intensities of LDHA bands from Western blot analysis were estimated by densitometric analysis. The results are presented as means ± SD. Data were statistically compared using the Student’s t-test. **p < 0.001 compared to the positive control (1^st^ column). **(C)** The binding of pyruvate to LDHA was determined using C^14^-labeled pyruvate. The rhLDHA was incubated with ^14^C-labeled pyruvate in absence or presence of PSTMB (0.5 μM). The non-bound ^14^C-pyruvate was washed out and the radioactivity was examined using a scintillation counter. Non-labeled pyruvate was used as the competitor. The results are presented as means ± SD. Data were statistically compared using the Student’s t-test. ***p < 0.001 compared to the negative control (1^st^ column). ^##^*p* < 0.01 compared with the control (2^nd^ column).

To elucidate the precise molecular mechanism underlying inhibition of LDHA activity by PSTMB, biochemical studies were performed. As LDHA forms a homotetrameric complex (LDH5) in order to convert pyruvate to lactate^20^, the inhibition of PSTMB on tetramer formation of LDHA was examined. The result clearly showed that PSTMB did not affect the tetramer formation of LDHA (Fig. 2A). In addition, the conversion of pyruvate to lactate is coupled to oxidation of the cofactor NADH to NAD^+^. Several compounds, including FX11, gossypol, and quinoline 3-sulfonamides, inhibited LDHA activity in an NADH-competitive manner^21–23^. Thus, we examined whether PSTMB inhibits the binding of NADH to LDHA using Cibacron Blue as an NADH mimic^24^. The result showed that PSTMB did not inhibit the interaction of Cibacron Blue with LDHA, whereas the binding was clearly inhibited by addition of NADH (Fig. 2B).

**Figure 3:**
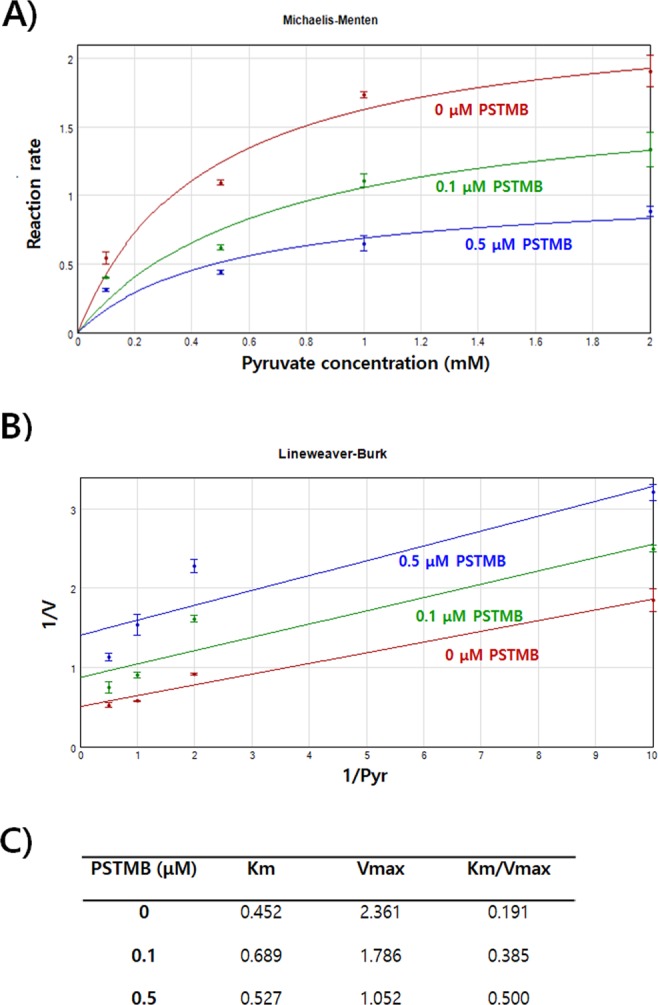
PSTMB inhibits LDHA activity in Michaelis-Menten and Lineweaver-Burk plots. To check LDHA enzyme kinetics, purified recombinant human LDHA protein (10 ng) were incubated in buffer containing 20 mM of HEPES-K^+^ (pH 7.2), 30 μM of NADH with 0, 0.1, 0.5, 1 and 2 mM of pyruvate in the presence or absence of PSTMB (0, 0.1, 0.5 μM). for 10 min. The fluorescence of NADH was examined at wavelength of excitation at 340 nm and emission at 460 nm with spectrofluorometer. Michaelis-Menten curves (**A**) and Lineweaver-Burk plots (**B**) are shown to determine the inhibition mode of PSTMB. The values are averages of three separate experiments. The results are plotted as means ± SD.

Oxamate, a pyruvate analogue, and small azoles harboring vicinal hydroxyl-carboxyl groups, such as 3-hydroxyisoxazole-4-carboxylic acid and 4-hydroxy-1,2,5-thiadiazole-3-carboxylic acid, have been shown to compete with pyruvate for binding to LDHA and thereby inhibit LDHA activity^20,25^. Similarly, the binding affinity of pyruvate to LDHA in the presence of PSTMB was examined using ^14^C radio-labeled pyruvate. The data clearly demonstrated that PSTMB inhibited the binding between radio-labeled pyruvate and LDHA (Fig. 2C). However, the structure of PSTMB is quite different from previously established pyruvate competitors, for example PSTMB has no hydroxyl-carboxyl group, which is regarded as essential for inhibitory activity^26^. Since PSTMB inhibited the binding of pyruvate to LDHA enzyme, the PSTMB inhibition mode was further confirmed by the Michaelis*–*Menten and Lineweaver Burk plots with a focus on pyruvate influence on PSTMB inhibition. PSTMB clearly decreased LDHA activity in a dose-dependent manner although LDHA activity was increased in a concentration of pyruvate-dependent manner (Fig. 3). Moreover, the corresponding of Lineweaver-Burks plots in Fig. 3B and values of *K*m and *V*max in Fig. 3C represented against the noncompetitive inhibition by PSTMB in the presence of pyruvate. PSTMB was also shown as a noncompetitive inhibitor, based on the Michaelis-Menten equation (Fig. 3A).

**Figure 4:**
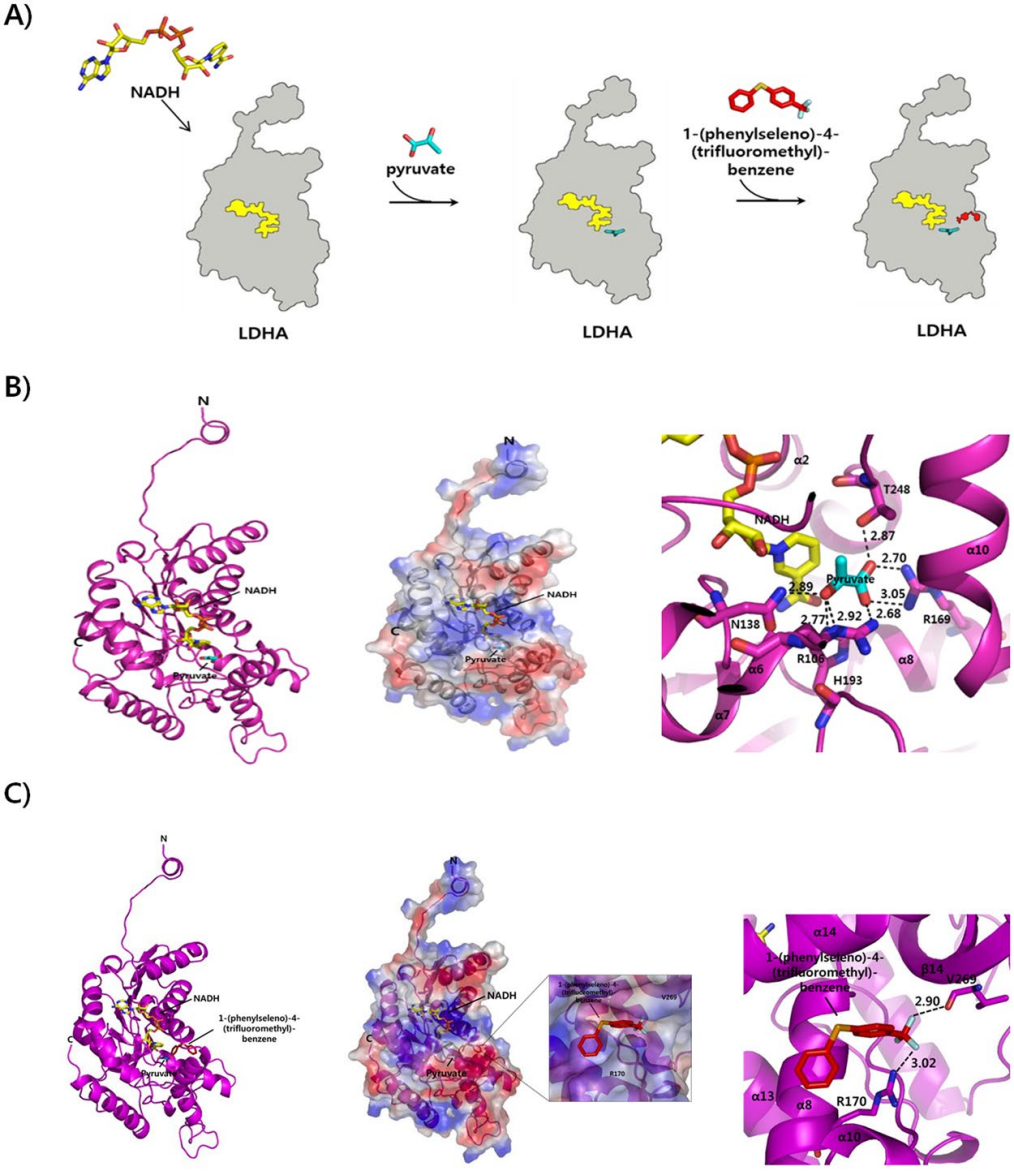
Formation of LDHA complex with NADH, pyruvate, or PSTMB and their overall modeled structures. **(A)** Schematic representations of LDHA complexed with NADH, pyruvate, or PSTMB are shown. **(B)** Ribbon and surface representations (left and middle panels) of the modeled structure of LDHA with NADH and pyruvate are shown. The interaction sites of the LDHA complex with NADH and pyruvate are shown (right panel). **(C)** The inhibitor PSTMB is bound to LDHA at the opposite site hole of the active site where pyruvate binds. The relative distribution of the surface charge is shown with the acidic region in red, basic region in blue and neutral region in white (middle panel).

An in-silico docking validation study was performed to check the docking scores of active compounds to LDHA with or without NADH (Table 1). We found that PSTMB had a lower binding free energy than the known active compounds oxamate or pyruvate^27,28^. In other words, PSTMB can bind to LDHA protein more efficiently than oxamate or pyruvate and thereby inhibit its activity, which is not depended on binding NADH cofactor to LDHA, because the docking score was almost similar for PSTMB binding to free LDHA (−6.0 kcal/mol) vs. NADH bound LDHA (−5.9 kcal/mol). Based on biochemical assays and computational modeling (Figs 2–4), it has been suggested that PSTMB may be an allosteric inhibitor of LDHA, which modify the pyruvate binding site due to imposed conformational changes to the LDHA enzyme for noncompetition inhibition.

**Table 1:** Docking scores of active compounds to LDHA with/without NADH.

Compounds	Docking Score (kcal/mol)	
	LDHA with NADH	LDHA without NADH
Pyruvate	−3.8	−3.5
Oxamate	−4.5	−4.1
PSTMB	−5.9	−6.0

### Architecture of the PSTMB inhibitor in LDHA activation by molecular modeling

LDHA is a promising molecular target for the treatment of various cancers^29^. For development of novel anti-cancer drugs that effectively inhibit LDHA activity, we performed molecular modeling of the binding interaction between human recombinant LDHA and selected inhibitor ligands. Through this study, the complicated biological action of the complex can be interpreted. The structural basis of LDHA with inhibitor as well as knowledge of the active site configuration and the catalytic mechanism can provide a means for discovery and structural optimization of the inhibitor. The three-dimensional structure of the LDHA has been previously identified^30^. LDHA is comprised of four subunits, each of which has an active site^18^. In this study, the structure of LDHA complex was modeled using the known structure of human LDHA (PDB ID: 1I10). Initial binding of the coenzyme NADH to the subunit was followed by binding of pyruvate (Fig. 4A). We modeled the structure of LDHA with NADH and pyruvate as a ribbon representation (Fig. 4B). Pyruvate binds to the residues (R106, N138, R169, H193, and T248) at the loop, α6 and α8 helices regions of LDHA.

To elucidate the role of the inhibitor PSTMB in LDHA activation, we constructed a structural model of the LDHA with NADH and inhibitor PSTMB and predicted their interaction sites (Fig. 3C). Inhibitor model building was initiated from the LDHA and NADH complex model and the inhibitor PSTMB was then included. Generally, the residues in the interaction sites of the complex had positive and negative charges in a globular fold. These charges may also promote formation of the target partner complex. We found that the negatively charged inhibitor PSTMB binds to the hydrophobic residue Val and the positively charged amino acid residues Arg of LDHA. LDHA is composed of twelve side-by-side α-helices and short ten β-sheets, and it has a hydrophilic pocket^30^. In our model (Fig. 4C), inhibitor PSTMB binds to the LDHA protein at the opposite site hole of the active site where pyruvate binds. Inhibitor PSTMB is located around α8 and β14 of the LDHA. In the complex structure of LDHA with NADH and inhibitor, the inhibitor PSTMB is seen bound to the residues R170 and V269 of LDHA. These two residues of LDHA were shown to play an important role in communication between LDHA and the inhibitor. The binding of the inhibitor was at a different orientation and angle compared with the binding of pyruvate to LDHA, and some conformational changes were likely induced by the binding of PSTMB. Thus, PSTMB inhibits the function of the LDHA and NADH complex by binding to the opposite site of active site where pyruvate binds.

### PSTMB inhibits the growth of cancer cells and intracellular LDHA activity

**Figure 5:**
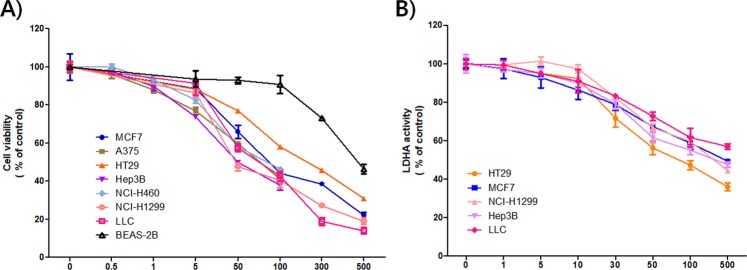
PSTMB inhibits cell viability and intracellular LDHA activity of various cancer cells. (**A**) The MCF-7, A375, HT29, Hep3B, NCI-460, NCI-H1299, LLC, BEAS-2B cells were treated with the indicated concentrations of PSTMB for 48 h. The viabilities of the cells were estimated by MTT assay. **(B)** The HT29, MCF-7, NCI-H1299, Hep3B, LLC cells were treated with indicated concentrations of PSTMB for 24 h. The cells were lysed and same concentration of the lysates were used for measuring LDHA activity.

As LDHA is a key enzyme for aerobic glycolysis, one of characteristic features of malignant tumors, it has been regarded as an attractive molecular target for cancer inhibition^4,18^. Genetic knockdown of LDHA expression or pharmacological inhibition of its activity suppressed the growth of diverse types of tumors^7,31,32^. Thus, the effect of PSTMB on growth of several cancer cell lines was examined. The results showed that PSTMB showed cytotoxic effect on several cancer cell lines of human or murine origin. However, in normal human bronchial epithelial BEAS-2B cells, the cytotoxic effect of PSTMB was limited (Table 2 and Fig. 5A).

**Table 2:** Cytotoxic effects of PSTMB on several tumor cell lines and normal cells.

Cell line	Origin	IC_50_ (μM)
NCI-H460	Human lung cancer cell	74.26 ± 2.17
NCI-H1299	Human lung cancer cell	45.33 ± 1.94
MCF-7	Human breast cancer cell	84.3 ± 1.92
Hep3B	Human hepatocellular carcinoma cell	45.33 ± 2.22
A375	Human melanoma cell	62.45 ± 2.24
HT29	Human colon cancer cell	73.34 ± 1.04
LLC	Mouse Lewis lung cancer cell	74.87 ± 1.22
BEAS-2B	Normal human bronchial epithelial cell	>300

**Figure 6:**
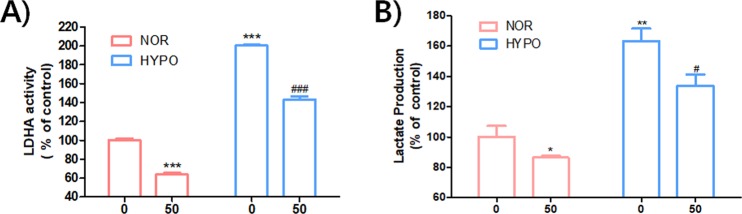
PSTMB inhibits LDHA activity and lactate production in HT29 cells under both normoxic and hypoxic conditions. The HT29 cells were cultured with the indicated concentrations of PSTMB with 20% (normoxia) or 0.1% (hypoxia) O_2_ for 24 h. **(A)** The cells were lysed and equal amounts of protein were used as enzyme source for LDHA activity assay. ***p < 0.001 compared with normoxia control (1^st^ lane) and ^###^*p* < 0.001 compared with hypoxia control (3^rd^ lane). **(B)** The culture media was changed with fresh media and cultured under the same condition for 1 h. Accumulated amounts of lactate in culture media were detected using the lactate assay kit. *p < 0.05, **p < 0.01 compared with normoxia control (1^st^ lane) and ^#^*p* < 0.05 compared with hypoxia control (3^rd^ lane).

Subsequently, the effect of PSTMB on LDHA activity in several cancer cells lines, such as human colon cancer HT29, human lung cancer NCI-H1299, human breast cancer MCF-7, human hepatocellular carcinoma Hep3B, and murine lung cancer LLC cells, was examined. The data clearly demonstrated that PSTMB could suppress intracellular LDHA activity in a dose-dependent manner (Fig. 5B). Under pathophysiological conditions like hypoxic microenvironments, the subsequent metabolic switch to an increased rate of fermentative glycolysis is a common affair to cancer cells^3,33^. The results from our study also showed that hypoxia increased both LDHA activity and lactate production. However, the hypoxia-induced LDHA activity and lactate production were clearly reduced by PSTMB treatment under both normoxic and hypoxic conditions (Fig. 6). These results collectively suggest that the suppression of LDHA by PSTMB was mainly mediated by the inhibition of enzyme activity, and not by the regulation of its expression.

### PSTMB Induced Mitochondrial ROS-Mediated Apoptosis

**Figure 7:**
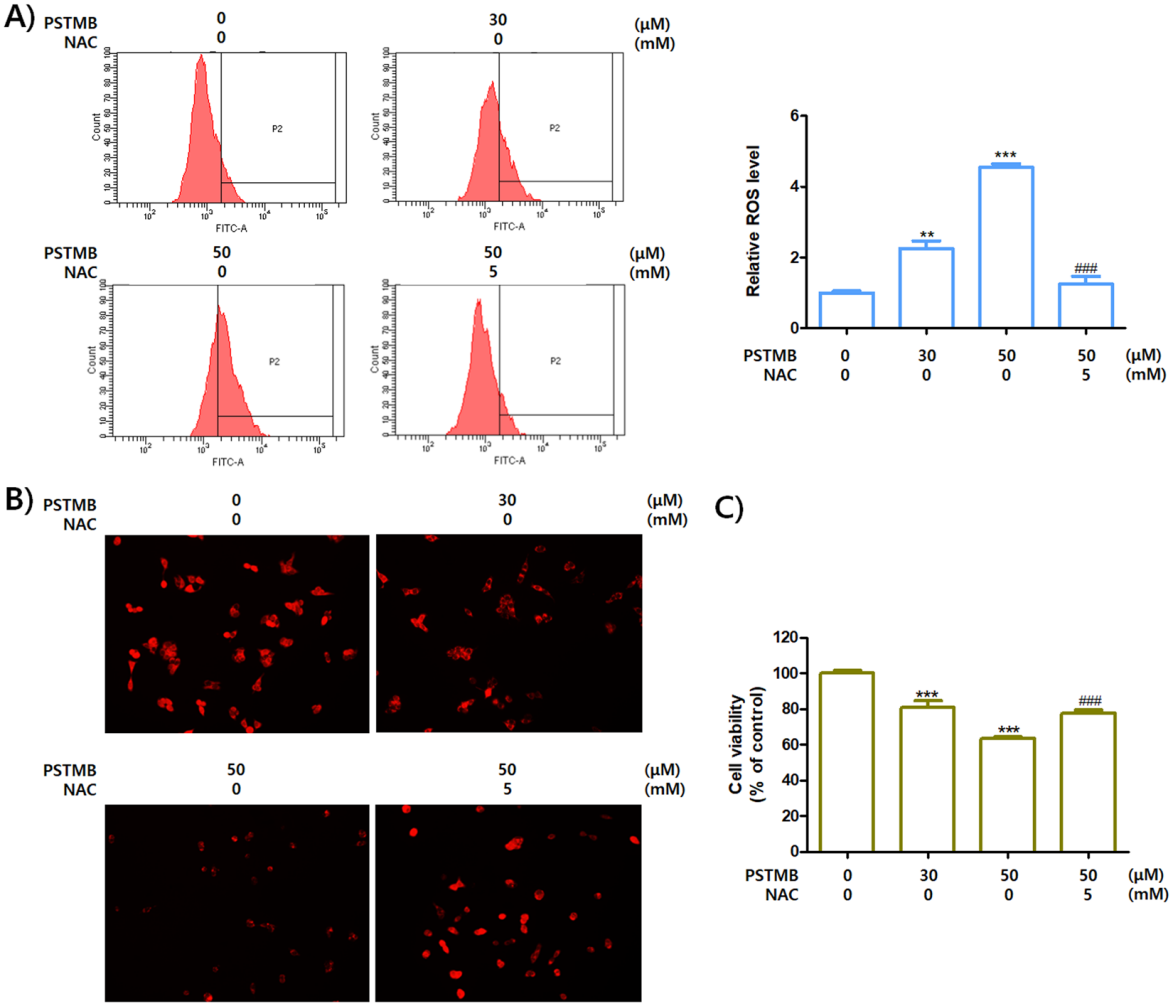
PSTMB induces ROS production and mitochondrial damage of HT29 cells. The HT29 cells were treated with indicated concentrations of PSTMB for 36 h, either in the presence or absence of a ROS-scavenger, NAC **(A)** The production of intracellular ROS was detected by FACS analysis using ROS indicator, H2DFCDA. The results are shown as mean ± SD. **p < 0.01 and ***p < 0.001 compared with control group. ^###^*p* < 0.001 compared with 3^rd^ lane. **(B)** The active mitochondria were stained with TMRM and pictured by fluorescence microscopy. The representative figures are shown. **(C)** The HT29 cells were treated with indicated concentrations of PSTMB for 36 h, in the presence or absence of NAC. The viabilities of the cells were estimated by MTT assay. ***p < 0.001 compared with control group and ^###^*p* < 0.001 compared with 3^rd^ lane.

**Figure 8:**
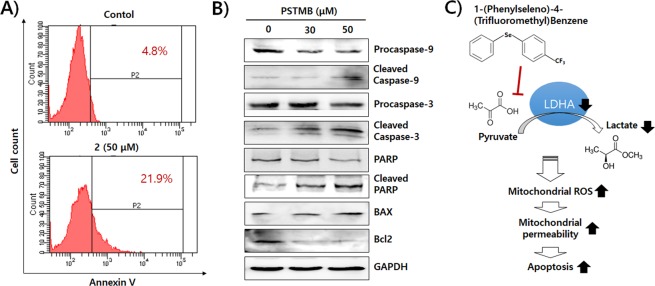
PSTMB induces apoptosis of HT29 cells. The HT29 cells were treated with indicated concentrations of PSTMB for 24 h. **(A)** The cells were harvested, stained with Annexin V/PI, and analyzed by flow cytometry. **(B)** The cells were lysed and the expression of proteins related with apoptotic pathway was examined by western blot analysis. **(C)** Schematic representation of the inhibitory effect of PSTMB on LDHA activity and tumor growth through mitochondrial ROS-mediated apoptosis.

As shown in Table 2, the growth rates of tumor cells were reduced by PSTMB treatment. Several previous studies demonstrated that pharmacological inhibition of LDHA activity or genetic knockdown of LDHA expression led to apoptotic cell death of cancer cells^21,31,34–36^. The mechanism involved in the induction of apoptosis by LDHA inhibition was verified as production of mitochondrial ROS^23,34^. The results shown in Fig. 7A,B demonstrate that PSTMB increases ROS generation and reduces the stability of the mitochondria. Treatment with the ROS scavenger, *N*-acetyl cysteine (NAC), reversed the PSTMB-induced ROS production and mitochondrial instability. In addition, NAC treatment rescued the death of HT29 cells induced by PSTMB treatment (Fig. 7C). Subsequently, the apoptotic changes induced by PSTMB treatment were examined. The data clearly shows that the population of Annexin V-positive HT29 cells increased in the presence of PSTMB in a dose-dependent fashion (Fig. 8A). Moreover, a marker of mitochondrial membrane stability, ratio of bcl-2/bax, was decreased by PSTMB treatment. The molecules involved in apoptosis cascade, such as caspase-9, caspase-3, and PARP, were also activated by PSTMB treatment (Fig. 8B). These results suggest that PSTMB induces the intrinsic pathway-mediated apoptosis of cancer cells via production of mitochondrial ROS (Fig. 8C).

## Discussion

ROS have paradoxical effects on progression and treatment of cancer. As increased ROS levels and altered redox status have been observed in almost all cancer cells, ROS are regarded as one of key tumor-promoting factors^37,38^. For example, ROS are involved in cell proliferation, cell cycle progression, cell survival, energy metabolism, cell motility, angiogenesis, and maintenance of tumor stemness^37^. The range of intracellular ROS achieved by the balance of ROS generation and ROS scavenging are important to the fate of tumor cells^39^. To promote redox signaling without excessive oxidant stress, tumor cells strongly depend on their elevated antioxidant defense system^40^. Although cancer cells generate increased ROS, these ROS levels are still below that which cause overt damage^41^. However, many chemotherapeutic agents have been designed to significantly increase the intracellular ROS levels in order to induce irreversible damages and subsequent apoptotic cell death^37,42^.

The mechanisms underlying the anti-cancer effects of these agents are often the induction of mitochondrial ROS production and inactivation of the antioxidant defense systems through metabolic inhibition^42–44^. Multiple alterations to the cellular metabolic pathways are linked to the synthesis of essential building blocks, such as amino acids, lipids, and nucleotides^45^. In addition, the substrates of these pathways are used to generate not only antioxidant molecules, including NADPH and glutathione (GSH), but also redox cofactors, such as NADH and FADH^43,46^. Among the cellular metabolic pathways involved in redox homeostasis, glycolysis is recognized as the essential player in the control of homeostasis in tumor, because glycolytic intermediates can be shuttled into metabolic pathways that generate reducing equivalents, such as NADPH and GSH^43^. Recent studies have shown that suppression of aerobic glycolysis by LDHA inhibitors, including FX11 and oxamate, impaired the progression of cancer through induction of oxidative stress^19,23^.

Furthermore, mitochondria is a major target for cancer therapy, since integrity loss of the outer mitochondrial membrane and subsequent release of proteins from the intermembrane space is one of the pivotal events in the apoptotic process^44^. However, resistance to mitochondrial permeabilization is common in cancer cells. The phenomenon often arises from upregulation of anti-apoptotic bcl-2 family proteins, subsequently blocking the permeability transition pore complex opening, or failure of pro-apoptotic bax/bak activation^47^. Moreover, it also arises from alterations in mitochondrial bioenergetics, i.e., shifting from mitochondrial oxidative phosphorylation toward cytoplasmic glycolysis^47,48^. Mitochondrial depolarization and increased ROS production are dependent on the flux of electrons from TCA cycle and oxidative phosphorylation^49^. Therefore, inhibiting entry of pyruvate into mitochondria is a promising strategy for cancer chemotherapy^47,50,51^. Based on these points of view, we suppose that the novel LDHA inhibitor, PSTMB, can be a potent candidate for development of anti-cancer therapeutic agents.

In conclusion, a novel selenobenzene, PSTMB, synthesized during our previous study, was found to be a potent inhibitor of the human LDHA enzyme. This is the very first report in scientific literature of a selenobenzene chalcogenide that can inhibit LDHA activity. Biochemical assays showed that PSTMB was noncompetitive with the binding of pyruvate to LDHA. Cellular assays demonstrated that PSTMB can suppress LDHA activity and production of lactate under both normoxia and hypoxia. It reduced cancer cell proliferation through induction of mitochondrial ROS production, loss of mitochondrial membrane integrity, and subsequent increased induction of apoptotic cell death. From these results, we conclude that PSTMB can provide an alternate option for inhibiting LDHA activity and for targeting glucose metabolism as an anti-tumor strategy.

## Methods

### General Information

Selenobenzene compounds used in this experiment were synthesized and structurally confirmed in our previous study^11^. The purities of these compounds are over 98%, measured by GC-MS analysis. The interference results of active compounds on LDHA activity, such as 1-(phenylseleno)-4-(trifluoromethyl) benzene (PSTMB), 1-methyl-4-phenylselenobenzene, 1-methoxy-4-(phenylseleno)benzene, 4-(phenylseleno)-1,1′-biphenyl, tetrahydro-3-(phenylseleno) thiophene, and 1-methoxy-4-[(phenylmethyl)seleno]benzene, were searched using Pan Assay Interference Compounds (PAINS) system^17^. Anti-LDHA antibody was obtained from Abcam (Cambridge, MA, USA). Antibodies for detecting caspase-3, caspase-9, and poly ADP-ribose polymerase (PARP) were purchased from Cell Signaling Technology. Antibodies against bcl-2 and bax were purchased from Novus Biologicals (Littleton, CO, USA). Antibody against GAPDH was purchased from Santa Cruz Biotechnology (Santa Cruz, CA, USA). All other commercial reagents, including 3-(4,5-dimethylthiazol-2-yl)-2,5-diphenyltetrazolium bromide (MTT) and tetramethylrhodamine methyl ester (TMRM), were provided by Sigma-Aldrich (St. Louise, MO, USA).

#### Selenobenzene compounds for assays

Generally, various selenium compounds required dissolution in DMSO before adding to the medium or buffer for each assay^52,53^. Usually, final concentration of DMSO in medium is from 0.1% to 0.5%. For test selenobenzene compounds of limited aqueous solubility, samples required dissolution in DMSO before adding to the medium or buffer for each assay, final concentration of DMSO in the cell culture medium or for each assay was 0.1% (v/v).

### *In vitro* LDH Activity Assay

For LDHA activity, the amounts of consumed NADH were measured^54^. Briefly, the indicated concentrations of PSTMB were incubated in buffer containing 20 mM of HEPES-K^+^ (pH 7.2), 20 μM of NADH, 2 mM of pyruvate, and 10 ng of purified recombinant human LDHA protein for 10 min. The fluorescence of NADH, which has an excitation wavelength of 340 nm and emission wavelength of 460 nm, was detected using a spectrofluorometer (Spectramax M2; Molecular Devices, Sunnyvale, CA, USA).

### Glutaraldehyde Cross-Linking Assay

In order to determine whether the LDHA protein exists as a monomer or oligomer after adding PSTMB, glutaraldehyde cross-linking of the LDHA protein was carried out. PSTMB in 20 mM HEPES reaction buffer (pH 8.0) and 10 μg of purified LDHA were incubated with 0.001–0.01% glutaraldehyde. The reaction was allowed to proceed for 2–5 min at 37 °C and then stopped with the addition of 1M Tris-HCl (pH 7.0) for 10 min at room temperature. The crosslinked products were analyzed by 15% SDS-PAGE followed by Coomassie blue staining.

### NADH binding ability Assay

NADH binding ability assay was performed as previously described^55^. The NADH binding ability of LDHA was determined by measuring the affinity of LDHA to agarose-immobilized Cibacron Blue 3GA, which mimics NADH^24^. Purified LDHA (400 ng) was incubated with NADH or PSTMB, followed by incubation with 30 µl of Cibacron Blue agarose at 4 °C for 2 h. After a washing step with 20 mM Tris-HCl (pH 8.6), LDHA bound to beads was eluted in PBS with SDS gel running buffer and subjected to SDS-PAGE, followed by western blotting. The same amount of protein was loaded as input to ensure equivalent protein amounts in every reaction.

### Pyruvate Binding Assay

For Pyruvate binding assay, 50 μg of purified His tagged-LDHA and pre-equilibrated Ni-NTA beads (Amersham Pharmacia Biotech, Little Chalfont, UK) were allowed to react for 30 min by rotating at 4 °C. Beads were centrifuged at 805 g for 3 min and washed three times with buffer A [20 mM HEPES-K^+^ (pH7.2) and 0.05% BSA]. Proteins bound to the beads were incubated with 5 μCi [^14^C] Pyruvate and PSTMB/sodium pyruvate for 5 min at 37 °C in a buffer A. The beads were then washed three times with 0.5 mL of ice-cold Buffer A. The bead-bound LDHA proteins were then eluted [50 mM Tris-HCl (pH 8.0), 200 mM imidazole], and radioactivity was analyzed by liquid scintillation counting using a Tri-Carb 3110TR System Liquid scintillation analyzer (PerkinElmer, Waltham, MA, USA) with the scintillation cocktail (ULTIMA GOLD AB™; PerkinElmer).

### Prediction of Protein-Small Molecule Interaction

Model of LDHA was constructed using SWISSMODEL software, a program for relative protein structure modeling. The result of an ExPASy search with the PDB ID revealed a reference protein: LDHA-NADH (PDB ID: 1I10). The 2D structure of PSTMB was obtained from the NCBI PubChem Compound database. The ID of PSTMB is CID_10494496. The 2D structure of PSTMB was converted to energy minimization of the structure by using OpenBabel in Pyrx. The prediction of LDHA protein and inhibitor PSTMB complex structure was performed using Autodock vina in Pyrx. Analysis of protein and small molecule docking generated by the Autodock vina program was modified with PyMOL. Authors will release the atomic coordinates and experimental data upon article publication.

### Cell Culture

The human colon cancer HT29 cells, hepatocellular carcinoma Hep3B cells, breast cancer MCF-7 cells, large cell lung cancer NCI-H460 cells, lymph node metastasized lung cancer NCI-H1299 cells, normal human bronchial epithelial BEAS-2B cells, and murine Lewis lung carcinoma (LLC) cells were obtained from the American Type Culture Collection (ATCC; Rockville, MD, USA). The HT29, Hep3B, MCF-7, BEAS-2B, and LLC cells were cultured in Dulbecco’s Modified Eagle Medium (DMEM; Welgene, Daegu, Korea) supplemented with 10% heat-inactivated fetal bovine serum (FBS; Sigma-Aldrich) and 1% penicillin/streptomycin (Gibco, Rockville, MD, USA). The NCI-H460 and NCI-H1299 cells were cultured with Roswell Park Memorial Institute 1640 (RPMI1640; Welgene) containing 10% heat-inactivated FBS and 1% penicillin/streptomycin. All cells were cultured at 37 °C in an atmosphere containing 5% CO_2_/air.

### Cell Viability Assay

The cells were cultured in 24-well plates with the indicated concentrations of PSTMB in serum-free media for 24 or 48 h. The media were then replaced with MTT solution (2 mg/mL) and incubated at 37 °C in a cell culture incubator for 3 h. The formed formazan crystals were fused with dimethyl sulfoxide and ethanol solutions. The viabilities of cells were estimated by measuring the absorbance at 540 nm using a spectrofluorometer.

### Intracellular LDH Activity Assay

The LDH activities from the lysates of cells incubated with indicated concentrations of PSTMB were determined by measuring the decrease in fluorescence caused by oxidation of NADH. Briefly, the total protein from cell lysates (1 μg) were mixed with 20 mM HEPES-K^+^ (pH 7.2), 0.05% BSA, 20 μM NADH, and 2 mM pyruvate (Sigma-Aldrich). The absorbance was measured using spectrofluorometer at an excitation wavelength of 340 nm and emission wavelength of 460 nm.

### Western Blot Analysis

Total protein was isolated from HT29 cells using 1% NP-40 lysis buffer [150 mM NaCl, 10 mM HEPES (pH 7.45), 1% NP-40, 5 mM NaPyrophosphate, 5 mM NaF, 2 mM Na_3_VO_4_] containing protease inhibitor cocktail tablet (Roche, Mannheim, Germany). Equal amounts (20 μg) of protein were separated by SDS-PAGE and electro-transferred to nitrocellulose membranes (Bio-Rad Laboratories, Hercules, CA, USA). The membranes were blocked by 5% non-fat skim milk (Sigma-Aldrich) in TBS, washed twice with TBS, and incubated with primary antibodies for target proteins at 4 °C overnight. Then, the membranes were washed three times with TBS and incubated with appropriate secondary antibodies conjugated with horseradish peroxidase. The specific bands of the proteins of interest were detected using ECL Plus (GE Healthcare) and chemiluminescence imaging system (ImageQuant LAS 4000; GE Healthcare).

### Induction of Hypoxia

For induction of hypoxia condition (1% oxygen), the cells were cultured in a mixture of 94% nitrogen and 5% CO_2_/air at 37 °C for 24 h in a cell culture incubator (Astec, Tokyo, Japan).

### Determination of Lactate Production

The cells were treated with the indicated concentrations of PSTMB in serum-free DMEM for 24 h. To accumulate lactate produced by the cells, the culture media were changed to phenol red- and serum-free DMEM for 1 h. The amounts of lactate in the media were measured with a lactate fluorometric assay kit (Biovision, Milpitas, CA, USA).

### Measurement of ROS

The intracellular ROS was detected using carboxy-H2DCFDA (5-(and-6)-carboxy-2′,7′-dichlorodihydrofluorescein diacetate; Thermo Fisher Scientific). The HT29 cells were treated with the indicated concentrations of PSTMB for 36 h, in the presence or absence of a ROS scavenger, *N*-acetyl cysteine (NAC). The cells were incubated with 100 μM carboxy-H2DCFDA at 37 °C for 30 min and washed with PBS.

### Determination of Mitochondrial Membrane Potential

The HT29 cells were treated with indicated concentrations of PSTMB for 36 h, in presence or absence of NAC. Next, treated cells were incubated with TMRM (Thermo Fisher Scientific) for 1 h at 37 °C and then washed with PBS twice. The samples were fixed with 3.7% formaldehyde and visualized by fluorescence microscopy (Axioimager M1 microscope, Zeiss, Aalen, Germany).

### Detection of Apoptotic Cells

HT29 cells were treated with the indicated concentrations of PSTMB for 36 h. Apoptotic cells were detected using the Annexin V-FITC Apoptosis Detection kit (Life Technologies, Carlsbad, CA, USA). The cells were suspended in 500 μL of buffer and treated with 5 μL of Annexin V-FITC. The fluorescence intensities were measured using BD FACSCANTO II (BD Biosciences, Franklin Lakes, NJ, USA).

### Statistical Analysis

The values from LDHA activity, pyruvate binding, cell viability, and lactate production assays were calculated as a percentage of the control and expressed as mean ± SEM. The levels of ROS are presented as fold-change over control. The statistical differences between groups were estimated by one-way analysis of variance (one-way ANOVA) and student’s t-test using GraphPad Prism software (GraphPad Software, La Jolla, CA, USA). All the experiments were performed at least 3 times, independently.

## Supplementary information

Supplementary Data

## Data Availability

All data generated or analyzed during this study are included in this published article (and its Supplementary Information Files).
