# Supplementary material for: A Novel Lactate Dehydrogenase Inhibitor, 1-(Phenylseleno)-4-(Trifluoromethyl) Benzene, Suppresses Tumor Growth through Apoptotic Cell Death
Source: Sci Rep. 2019 Mar 8;9:3969. doi: 10.1038/s41598-019-40617-3 (PMC6408513; doi:10.1038/s41598-019-40617-3)
Supplement: Supplementary file 1 — Supplementary Data [file 41598_2019_40617_MOESM1_ESM.doc]

**Supplementary information**

**A Novel Lactate Dehydrogenase Inhibitor, 1-​(Phenylseleno)​-​4-​(Trifluoromethyl)​Benzene, Suppresses Tumor Growth through Apoptotic Cell Death**

Eun-Yeong Kim^1, †^, Tae-Wook Chung^1, †^, Chang Woo Han^2^, So Young Park^2^, Kang Hyun Park^3^, Se Bok Jang^2, *^, and Ki-Tae Ha^1, *^

^1^Department of Korean Medical Science, School of Korean Medicine and Healthy Aging Korean Medical Research Center, Pusan National University, Yangsan, Gyeongnam 50612, Republic of Korea

^2^Department of Molecular Biology and ^3^Department of Chemistry, College of Natural Science, Pusan National University, Geumjeong-gu, Busan 46241, Republic of Korea

*Correspondence to: [sbjang@pusan.ac.kr](mailto:sbjang@pusan.ac.kr) (S.B.J.) and [hagis@pusan.ac.kr](mailto:hagis@pusan.ac.kr) (K.T.H.)

^]†^These authors contributed equally to this work.


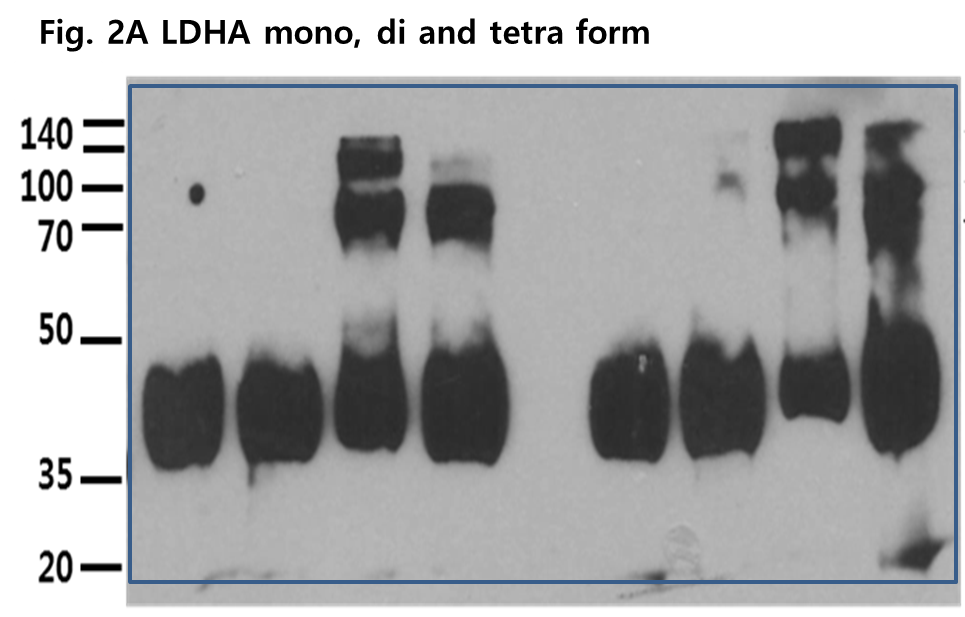

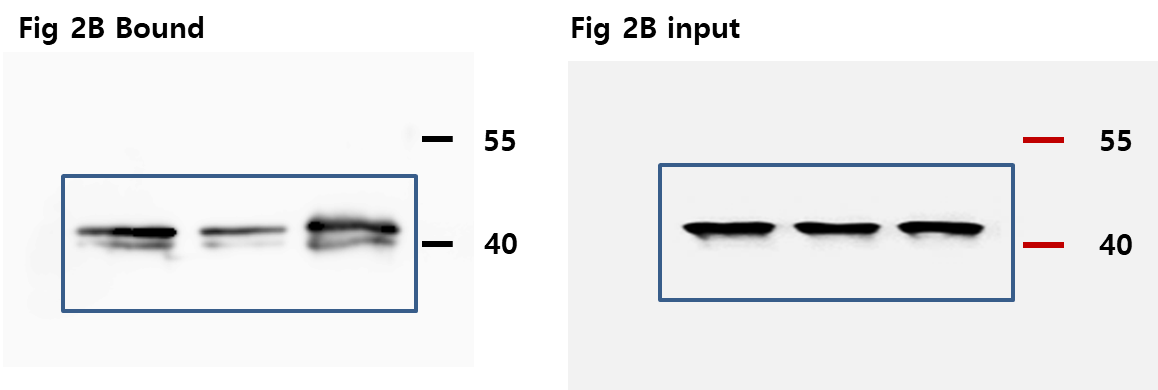


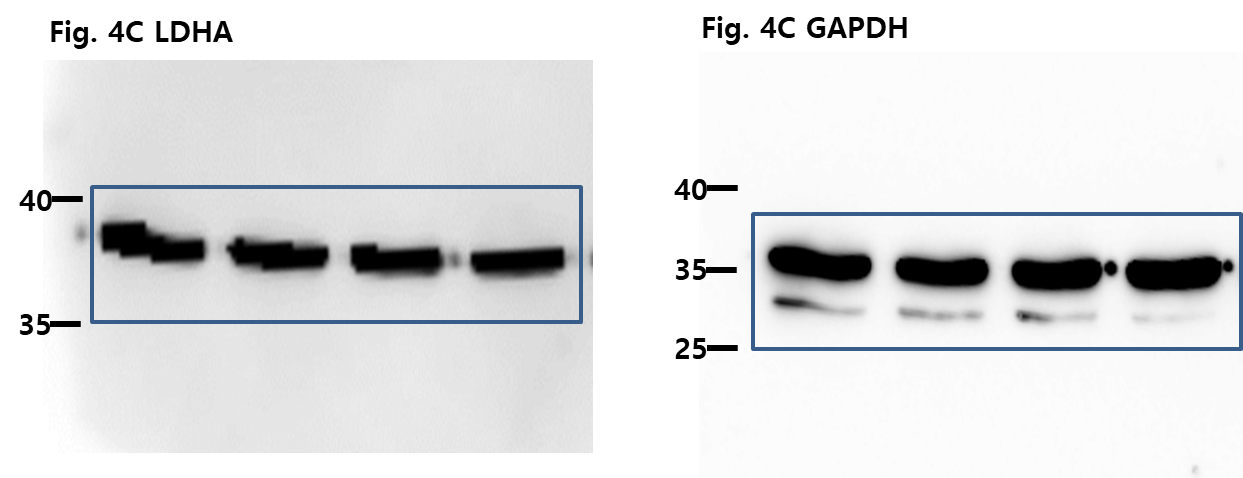


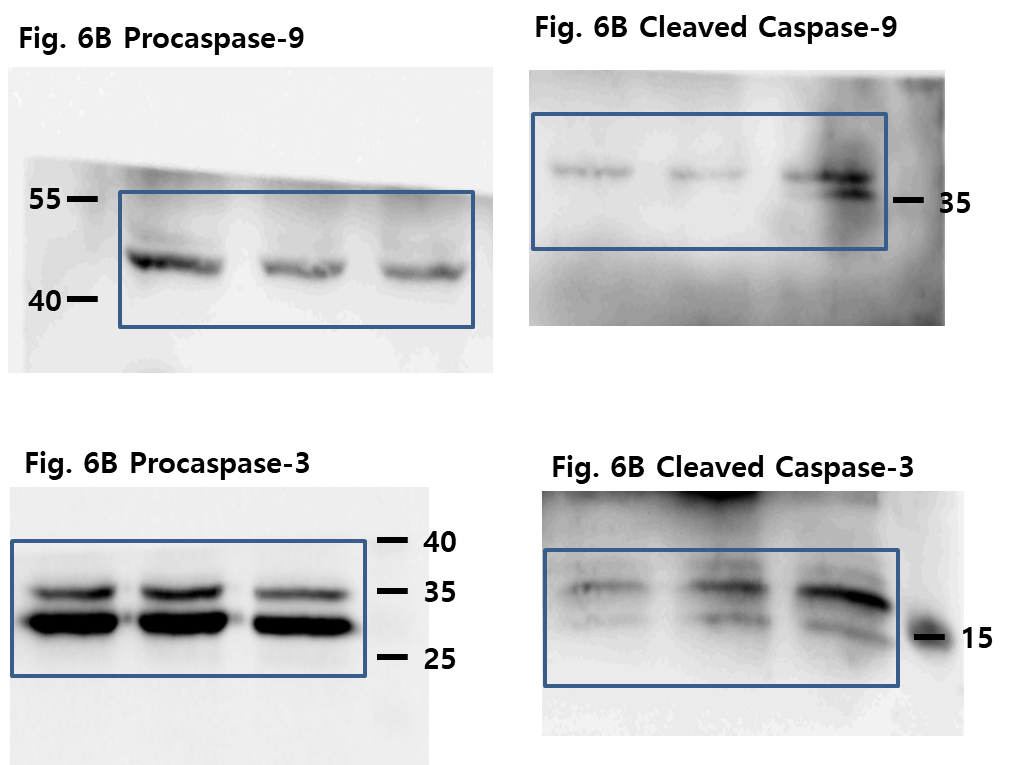

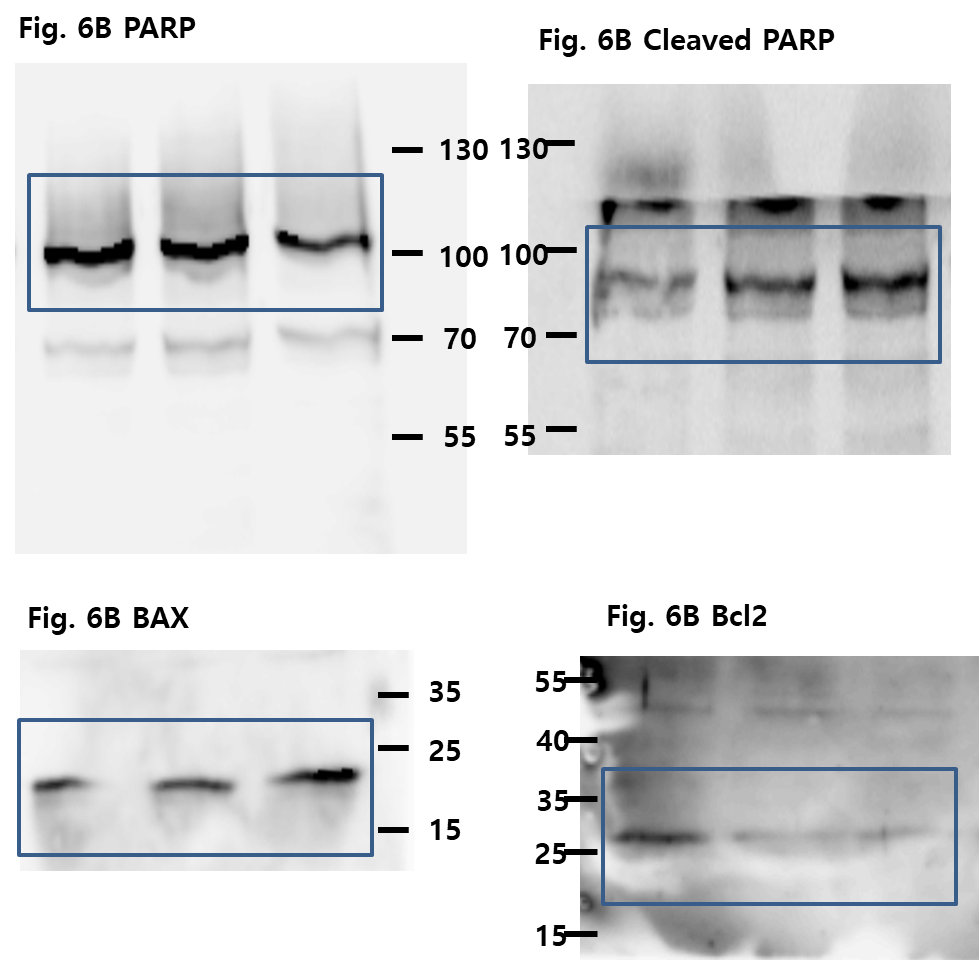


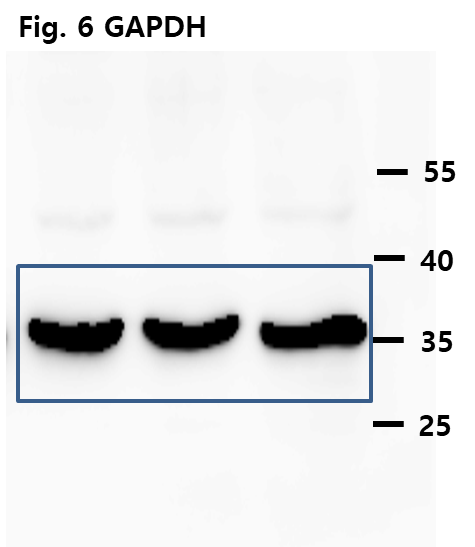


**Figure S1. Uncropped immunoblots of the most important blots.**
